# Supplementary material for: Selective ablation of VIP interneurons in the rodent prefrontal cortex results in increased impulsivity
Source: PLoS One. 2023 Jun 2;18(6):e0286209. doi: 10.1371/journal.pone.0286209 (PMC10237669; doi:10.1371/journal.pone.0286209)
Supplement: S1 Fig — (A) Representative images of the spread of caspase-3 AAV. PL = prelimbic, IL = infralimbic, DP = dorsal peduncular cortex. (B) Quantitative analysis of ablation of VIP interneurons in the PL vs IL. Caspase ablation was localized to the IL, as indicated by a significant decrease of VIP interneurons (represented by ZsGreen expression) in the IL (pIL = 0.0254) but not in the PL (pPL = 0.1092). Additionally, there are significantly fewer VIP interneurons in the IL of caspase animals (pcaspase = 0.0499) but not in the sham animals (psham = 0.8284), indicating specific ablation of VIP interneurons in the IL. (DOCX) [file pone.0286209.s001.docx]

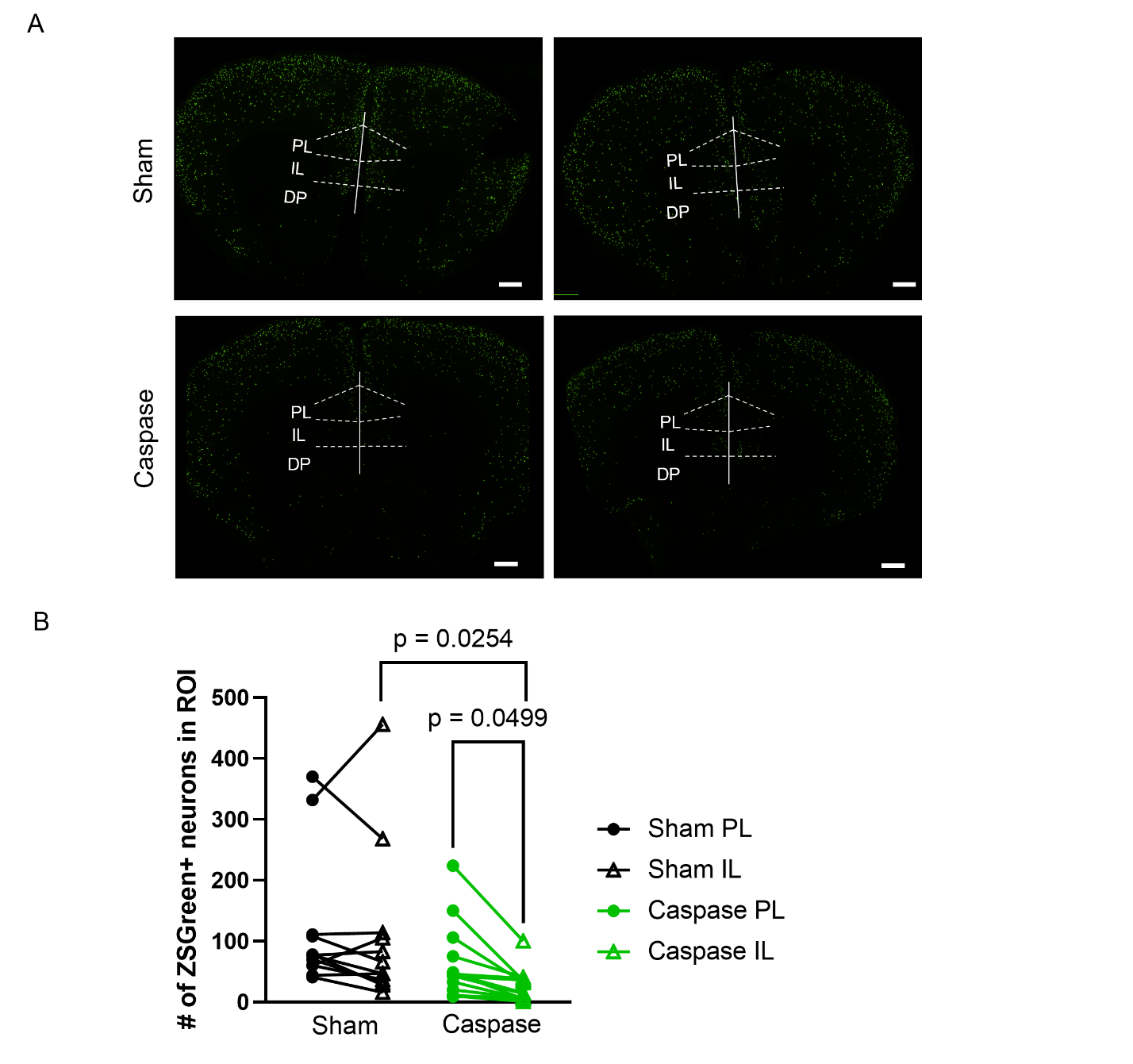


**S1 Fig. Caspase ablation of VIP interneurons is primarily localized to the IL.** (A) Representative images of the spread of caspase-3 AAV. PL = prelimbic, IL = infralimbic, DP = dorsal peduncular cortex. (B) Quantitative analysis of ablation of VIP interneurons in the PL vs IL. Caspase ablation was localized to the IL, as indicated by a significant decrease of VIP interneurons (represented by ZsGreen expression) in the IL (p_IL_ = 0.0254) but not in the PL (p_PL_ = 0.1092). Additionally, there are significantly fewer VIP interneurons in the IL of caspase animals (p_caspase_ = 0.0499) but not in the sham animals (p_sham_ = 0.8284), indicating specific ablation of VIP interneurons in the IL.
