# Supplementary material for: Selective ablation of VIP interneurons in the rodent prefrontal cortex results in increased impulsivity
Source: PLoS One. 2023 Jun 2;18(6):e0286209. doi: 10.1371/journal.pone.0286209 (PMC10237669; doi:10.1371/journal.pone.0286209)
Supplement: S2 Fig — (A) Caspase ablation results in a trend of increased premature responding in both male and female mice, with a stronger increase in female animals, while not significant. No significant differences between male and female animals. (B) ITI length corresponds to an increase in premature responses in both males and females but is not significant. No significant differences between male and female animals. Statistics summarized in S1 and S2 Tables. (DOCX) [file pone.0286209.s002.docx]

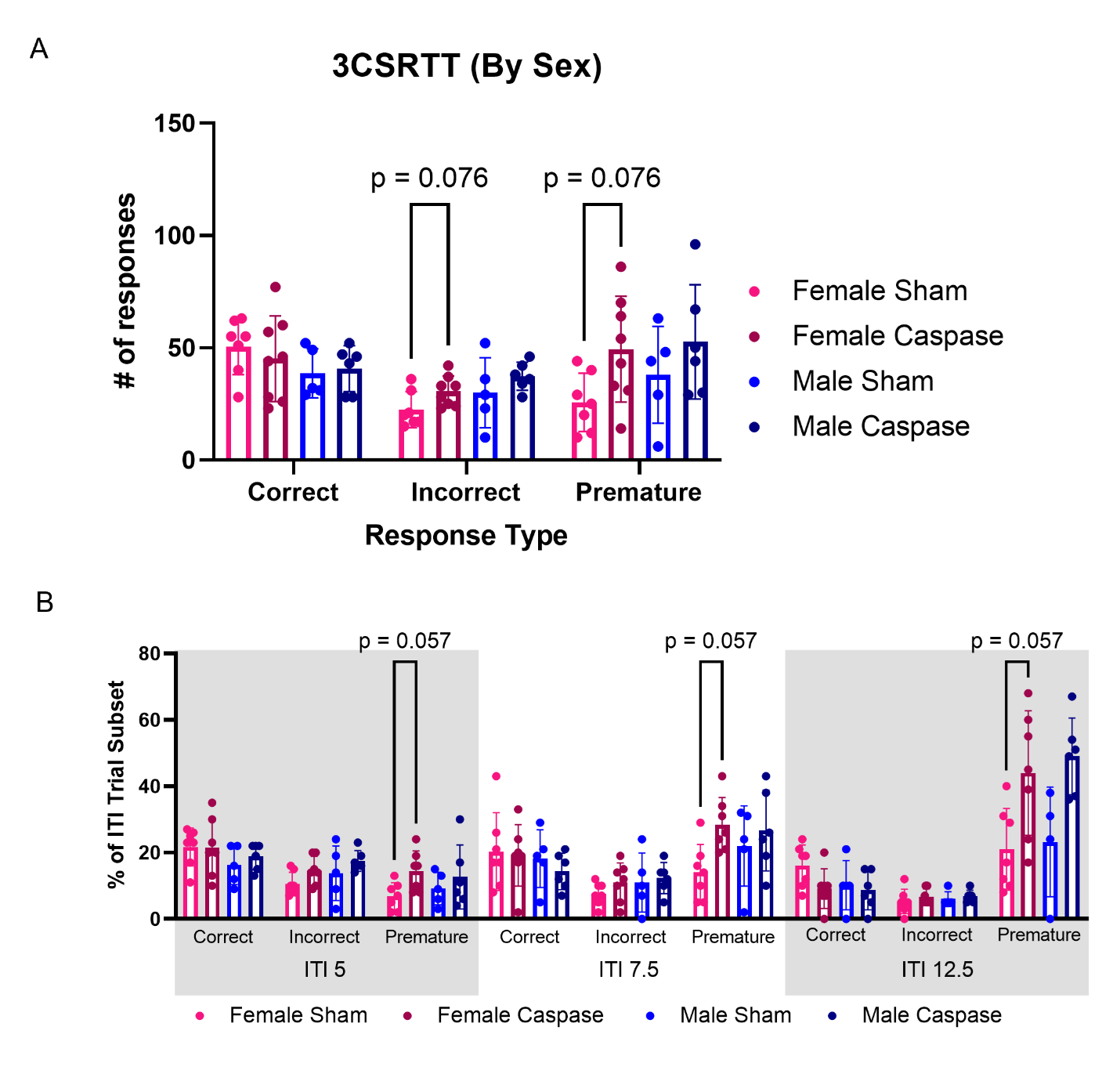


**S2 Fig. Caspase ablation of IL VIP interneurons does not cause discriminate impulsive behavior in males vs females.** (A) Caspase ablation results in a trend of increased premature responding in both male and female mice, with a stronger increase in female animals, while not significant. No significant differences between male and female animals. (B) ITI length corresponds to an increase in premature responses in both males and females but is not significant. No significant differences between male and female animals. Statistics summarized in S1 Table and S2 Table.
