# Supplementary material for: Selective ablation of VIP interneurons in the rodent prefrontal cortex results in increased impulsivity
Source: PLoS One. 2023 Jun 2;18(6):e0286209. doi: 10.1371/journal.pone.0286209 (PMC10237669; doi:10.1371/journal.pone.0286209)
Supplement: S3 Fig — (A) Caspase ablation does not affect novelty-seeking behavior as measured by novel social assay in either males or females. No significant differences between male and female animals. (B) Caspase ablation does not affect anxiety-related behavior as measured by time spent in center of open field and overall locomotion in either males or females. No significant differences between male and female animals. (C) Caspase ablation does not affect binge-like food intake in either males or females. No significant differences between male and female animals. Statistics summarized in S3 Table. (DOCX) [file pone.0286209.s003.docx]

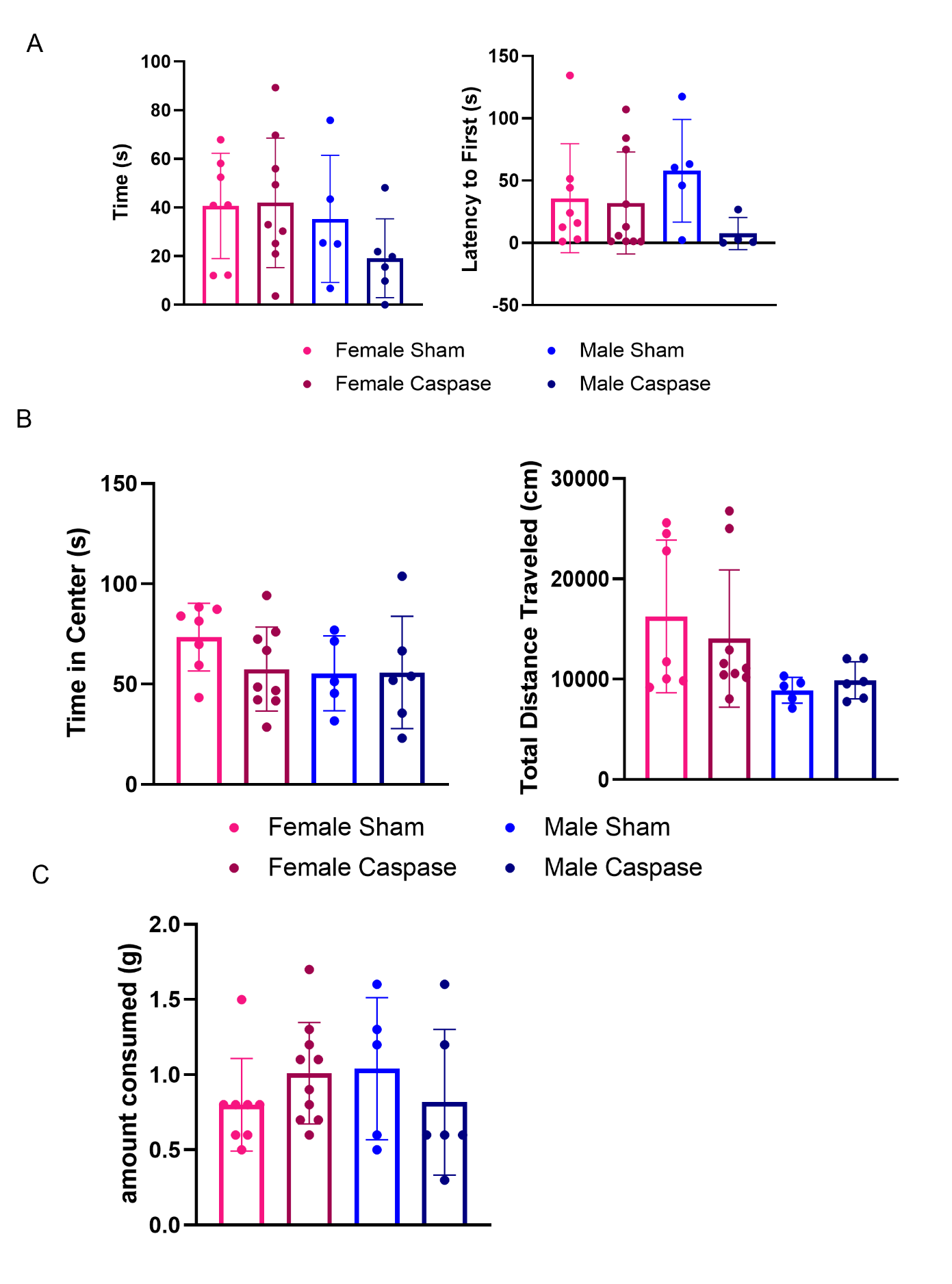


**S3 Fig. Caspase ablation of IL VIP interneurons does not cause discriminate novelty-seeking or anxiety-related behaviors in males vs. females.** (A) Caspase ablation does not affect novelty-seeking behavior as measured by novel social assay in either males or females. No significant differences between male and female animals. (B) Caspase ablation does not affect anxiety-related behavior as measured by time spent in center of open field and overall locomotion in either males or females. No significant differences between male and female animals. (C) Caspase ablation does not affect binge-like food intake in either males or females. No significant differences between male and female animals. Statistics summarized in S3 Table.
