# Supplementary material for: Selective ablation of VIP interneurons in the rodent prefrontal cortex results in increased impulsivity
Source: PLoS One. 2023 Jun 2;18(6):e0286209. doi: 10.1371/journal.pone.0286209 (PMC10237669; doi:10.1371/journal.pone.0286209)
Supplement: S1 Table — (DOCX) [file pone.0286209.s004.docx]

**S1 Table. Statistics summary for supplemental figure 1A.**

| **3-way ANOVA** |  |  |  |  |  |
| --- | --- | --- | --- | --- | --- |
|  | Sum of Squares | df | Mean Square | F (DFn, DFd) | P value |
| Response Type | 2557 | 2 | 1279 | F (2, 69) = 5.332 | P=0.0070 |
| Sex | 84.62 | 1 | 84.62 | F (1, 69) = 0.3529 | P=0.5544 |
| Treatment | 1416 | 1 | 1416 | F (1, 69) = 5.906 | P=0.0177 |
| Response Type x Sex | 988.8 | 2 | 494.4 | F (2, 69) = 2.062 | P=0.1350 |
| Response Type x Treatment | 1422 | 2 | 711 | F (2, 69) = 2.965 | P=0.0582 |
| Sex x Treatment | 5.565 | 1 | 5.565 | F (1, 69) = 0.02321 | P=0.8794 |
| Response Type x Sex x Treatment | 231.7 | 2 | 115.8 | F (2, 69) = 0.4830 | P=0.6190 |
| **Multiple unpaired t-tests with Welch's correction and FDR** |  |  |  |  |  |
| *Group 1* | Female Sham | Female Sham | Male Sham | Male Caspase |  |
| *Group 2* | Female Caspase | Male Sham | Male Caspase | Female Caspase |  |
| Correct | p = 0.455 | p = 0.338 | p = 0.764 | p = 0.800 |  |
| Incorrect | p = 0.045 | p = 0.364 | p = 0.557 | p = 0.534 |  |
| Premature | p = 0.045 | p = 0.364 | p = 0.557 | p = 0.800 |  |
