# Supplementary material for: Selective ablation of VIP interneurons in the rodent prefrontal cortex results in increased impulsivity
Source: PLoS One. 2023 Jun 2;18(6):e0286209. doi: 10.1371/journal.pone.0286209 (PMC10237669; doi:10.1371/journal.pone.0286209)
Supplement: S2 Table — (DOCX) [file pone.0286209.s005.docx]

**S2 Table. Statistics summary for supplemental figure 1B.**

| **3-way ANOVA** |  |  |  |  |  |
| --- | --- | --- | --- | --- | --- |
|  | Sum of Squares | df | Mean Square | F (DFn, DFd) | P value |
| Response Type | 13617 | 8 | 1702 | F (8, 206) = 23.62 | P<0.0001 |
| Sex | 43.29 | 1 | 43.29 | F (1, 206) = 0.6009 | P=0.4391 |
| Treatment | 906.5 | 1 | 906.5 | F (1, 206) = 12.58 | P=0.0005 |
| Response Type x Sex | 484.6 | 8 | 60.58 | F (8, 206) = 0.8408 | P=0.5677 |
| Response Type x Treatment | 3278 | 8 | 409.8 | F (8, 206) = 5.688 | P<0.0001 |
| Sex x Treatment | 1.101 | 1 | 1.101 | F (1, 206) = 0.01528 | P=0.9017 |
| Response Type x Sex x Treatment | 247.8 | 8 | 30.98 | F (8, 206) = 0.4299 | P=0.9022 |
| **Multiple unpaired t-tests with FDR** |  |  |  |  |  |
| *Group 1* | Female Sham | Female Sham | Male Sham | Male Caspase |  |
| *Group 2* | Female Caspase | Male Sham | Male Caspase | Female Caspase |  |
| Correct (ITI 5) | p = 0.789 | p = 0.593 | p = 0.703 | p = 0.995 |  |
| Incorrect (ITI 5) | p = 0.112 | p = 0.593 | p = 0.703 | p = 0.995 |  |
| Premature (ITI 5) | p = 0.113 | p = 0.593 | p = 0.703 | p = 0.995 |  |
| Correct (ITI 7.5) | p = 0.555 | p = 0.810 | p = 0.703 | p = 0.995 |  |
| Incorrect (ITI 7.5) | p = 0.295 | p = 0.593 | p = 0.764 | p = 0.995 |  |
| Premature (ITI 7.5) | p = 0.112 | p = 0.593 | p = 0.703 | p = 0.995 |  |
| Correct (ITI 12.5) | p = 0.112 | p = 0.593 | p = 0.764 | p = 0.995 |  |
| Incorrect (ITI 12.5) | p = 0.555 | p = 0.810 | p = 0.703 | p = 0.995 |  |
| Premature (ITI 12.5) | p = 0.112 | p = 0.810 | p = 0.173 | p = 0.995 |  |
