# Supplementary material for: Selective ablation of VIP interneurons in the rodent prefrontal cortex results in increased impulsivity
Source: PLoS One. 2023 Jun 2;18(6):e0286209. doi: 10.1371/journal.pone.0286209 (PMC10237669; doi:10.1371/journal.pone.0286209)
Supplement: S3 Table — (DOCX) [file pone.0286209.s006.docx]

**S3 Table. Statistics summary for supplemental figure 2.**

| **2-way ANOVA (Time Spent with novel animal)** | | |  | |  | |  | |  |
| --- | --- | --- | --- | --- | --- | --- | --- | --- | --- |
|  | Sum of Squares | df | | Mean Square | | F (DFn, DFd) | | P value | |
| Sex x Treatment | 486.8 | 1 | | 486.8 | | F (1, 23) = 0.8934 | | P=0.3544 | |
| Sex | 1270 | 1 | | 1270 | | F (1, 23) = 2.331 | | P=0.1405 | |
| Treatment | 355.7 | 1 | | 355.7 | | F (1, 23) = 0.6528 | | P=0.4274 | |
| **2-way ANOVA (Latency to approach novel animal)** | | |  | |  | |  | |  |
|  | Sum of Squares | df | | Mean Square | | F (DFn, DFd) | | P value | |
| Sex x Treatment | 3211 | 1 | | 3211 | | F (1, 23) = 2.065 | | P=0.1641 | |
| Sex | 8.729 | 1 | | 8.729 | | F (1, 23) = 0.005614 | | P=0.9409 | |
| Treatment | 4331 | 1 | | 4332 | | F (1,23) = 2.786 | | P=0.1086 | |
| **2-way ANOVA (Cumulative time in center of open field)** | | |  | |  | |  | |  |
|  | Sum of Squares | df | | Mean Square | | F (DFn, DFd) | | P value | |
| Sex x Treatment | 434.1 | 1 | | 434.1 | | F (1, 23) = 0.9471 | | P=0.3406 | |
| Sex | 627 | 1 | | 627 | | F (1, 23) = 1.368 | | P=0.2542 | |
| Treatment | 385.7 | 1 | | 385.7 | | F (1, 23) = 0.8414 | | P=0.3685 | |
| **2-way ANOVA (Total distance traveled)** | | |  | |  | |  | |  |
|  | Sum of Squares | Df | | Mean Square | | F (DFn, DFd) | | P value | |
| Sex x Treatment | 16245363 | 1 | | 16245363 | | F (1, 23) = 0.5010 | | P=0.4862 | |
| Sex | 214056079 | 1 | | 214056079 | | F (1, 23) = 6.602 | | P=0.0171 | |
| Treatment | 2301648 | 1 | | 2301648 | | F (1, 23) = 0.07099 | | P=0.7923 | |
| **Multiple unpaired t-tests with FDR (Total distance traveled)** | | |  | |  | |  | |  |
| Female Sham vs Male Sham | P = 0.0612 |  | |  | |  | |  | |
| Female Caspase vs Male Caspase | P = 0.1723 |  | |  | |  | |  | |
| **2-way ANOVA (Amount of HFD consumed)** | | |  | |  | |  | |  |
|  | Sum of Squares | df | | Mean Square | | F (DFn, DFd) | | P value | |
| Sex x Treatment | 0.3174 | 1 | | 0.3174 | | F (1, 25) = 2.116 | | P=0.1582 | |
| Sex | 0.0003005 | 1 | | 0.0003005 | | F (1, 25) = 0.002003 | | P=0.5393 | |
| Treatment | 0.003681 | 1 | | 0.003681 | | F (1, 25) = 0.02454 | | P=0.8768 | |
